# Manitoba

**Published:** 1896-04

**Authors:** 


### MANITOBA.

An Act to amend "The Veterinary Association Act," No. 24.

Her Majesty, by and with the advice and consent of the Legislative Assembly of the Province of Manitoba, enacts as follows:

1. Paragraph (c) of Section 4 of Chapter 148 of the Revised Statutes of Manitoba is hereby repealed and the following substituted therefor:

(c) Graduates of any recognized Veterinary School or College having a regular curriculum of not less than three sessions of six months each. Nothing in this paragraph shall affect or apply to persons holding or entitled to hold diplomas or certificates deemed satisfactory under the paragraph (c) for which this paragraph has been substituted, provided that such diplomas shall have been granted prior to the year 1898.

2. This Act shall come into force on the day it is assented to.

Remarks of members of the Legislature bearing upon the subject:

Mr. McNaught moved for a second reading of the bill to amend the Veterinary Association Act.

Mr. Crosby was opposed to the principles of the bill. This only served to make this incorporation the more close. He failed to see why gentlemen who may have come into the Province as graduates of two-years' colleges now wanted to keep out all but graduates of colleges with a three-year course. He wanted free trade in this as in anything else.

Mr. McNaught replied that this would protect the public by seeing that only properly qualified persons were allowed to practice. This kept out people who would collect money for services that were not worth anything.

Dr. Rutherford said that Mr. Crosby had an erroneous idea of the whole matter. By keeping the Act as it was it was not giving free trade, but in fact protecting one institution in Canada that was not giving a course of sufficient length. It was unpleasant to wash professional dirty linen in public, but he would do so. There was a gentleman in Toronto running what was called the Ontario Veterinary College, who had made half a million out of deluded youths who thought they could get a thorough veterinary education in two short terms. The way things were carried on at Toronto was simply disgraceful. There was only one other veterinary college in Canada, that in connection with McGill College, and here, in a three-year course, Dr. McEachran had labored to keep up the standard in Canada. All the larger and better colleges in the States were adopting three- or four-year courses. Great Britain, France, and Germany had long courses, and by the amended Act men from these good colleges would be allowed to come in, while the Ontario College would no longer have the monopoly. He compared the standing of the medical and veterinary professions in Canada. A graduate of one of our medical colleges could go to England and with six months' residence and passing the necessary examinations could become a member of the Royal College of Physicians and Surgeons; but a graduate of a veterinary college had to take the matriculation examination and go through the whole course. The result of this bill would simply be that the Toronto institution would be forced to add a third year to its course.

Mr. Sirett wanted all the improperly qualified veterinarians now in the Province to go back and take more rigid examinations.

Dr. Rutherford said this was an evil time alone would cure.

The bill was read and referred.

